# Milk beverages can reduce nutrient inadequacy among Brazilian pre-school children: a dietary modelling study

**DOI:** 10.1186/s40795-022-00620-w

**Published:** 2022-11-01

**Authors:** Yvonne M. Lenighan, Marie Tassy, Carlos A. Nogueira-de-Almeida, Elizabeth A. Offord, Tsz Ning Mak

**Affiliations:** 1 Department of Nutrition Sciences, Nestlé Institute of Health Sciences, Nestlé Research, Société des Produits Nestlé S.A., Vers-chez-les-Blanc, 1000 Lausanne, Switzerland; 2 Departamento de Medicina, Universidade Federal de São Carlos, São Carlos, SP Brazil

**Keywords:** Dietary modelling, Nutrient inadequacy, Dairy, Milk, Pre-school children

## Abstract

**Background:**

Previous studies found high prevalence of inadequate intakes of vitamins E, D and K, calcium and potassium among Brazilian pre-school children, with suboptimal consumption of dairy products. Dietary modelling was applied to determine the theoretical impact of improving dairy products consumption on nutrient adequacy in 4–5-year-old Brazilian children.

**Methods:**

Adherence to the dairy recommendation of two servings/day was calculated using data from the Brazil Kids Nutrition and Health Study (KNHS) (*n* = 228). Two modelling scenarios were applied to test the impact on nutrient intakes of (1) adding one or two servings of a frequently consumed cow’s milk or a widely available fortified alternative: pre-school children milk (PCM), and of (2) substituting the current milk consumed by PCM. Mean nutrient intakes and percentage of children adhering to the nutrient recommendations were determined at baseline and after applying modelling scenarios.

**Results:**

Seventy-six percent (*n* = 174) of children did not meet the recommended daily two servings of dairy products, 56% had less than one serving of dairy products on the day of recall. The mean consumption of whole milk (fortified and unfortified) was 147 g/d, yoghurt 114 g/d and cheese 34 g/d. The addition of one serving of cow’s milk demonstrated a 17% reduction in calcium inadequacy, 18% reduction in vitamin A and 3% reduction in zinc inadequacy. Adding one serving of PCM further reduced calcium inadequacy from 87 to 41%, vitamin E from 81 to 37%, and zinc inadequacy by 10%. Replacing the child’s current milk with a PCM resulted in further reduction of micronutrient inadequacies, including calcium, vitamin D and vitamin E.

**Conclusions:**

Dairy products consumption in pre-school children should be encouraged to reduce nutrient inadequacies. In particular, consumption of PCM would help to reduce calcium, vitamin D and vitamin E inadequacy, nutrients of concern in this population.

**Supplementary Information:**

The online version contains supplementary material available at 10.1186/s40795-022-00620-w.

## Background

Pre-school is a key period of growth and development, and a healthy balanced diet is required to support nutrient adequacy during this stage [1, 2]. In Brazil, the dietary pattern of infants and toddlers is characterized by low consumption of meat, fruits and vegetables; early and high intake of fried foods, candies/sweets, soft drinks and salt; and while consumption of unmodified cow’s milk is prevalent, the level of consumption is below the national recommendation of 2–3 servings of dairy products per day among children under 3 years [3–5]. A randomized controlled study examining the tracking of diet quality from pre-school to school-age children in Brazil showed that dietary patterns remained similar during this transition, except for milk and fruit consumption, that decreased over time [6]. In the recent Brazilian Kids Nutrition and Health Survey, it was observed that a high proportion of children 4 to 8 years had intakes below the Estimated Average Requirements or Adequate Intake thresholds for vitamins E, D and K; calcium, potassium and fiber [7]. Dairy products are a key source of calcium and other micronutrients, which are vital for growth [8], and regular consumption should be encouraged in pre-school children to improve nutrient adequacy.

Dietary modelling is a technique that has been applied for the past three decades to determine the theoretical impact of modifying the diet on nutrient adequacy, using dietary intake data and statistical techniques [9]. This technique has been commonly applied in the development of national dietary guidelines in a number of countries, including US, Ireland and Australia [10–12], in order to determine which foods should be added to, or removed from, diet, and the appropriate quantities required to improve nutrient adequacy in the population of interest. A recent publication from Dewey et al. [13] highlights the application of modelling in determining food pattern recommendations for infants and toddlers in the 2020–2025 dietary guidelines. Different models were applied, which incorporating the age of the infant, daily energy requirements, feeding type (e.g. breastfed vs formula-fed) and appropriate food groups to support a healthy, balanced diet. In the US infant and toddler population, diet modelling identified that a combination of fortified infant cereal and high protein foods would help to achieve nutrient adequacy [13]. A similar approach was applied in the Irish dietary guidelines, particularly with respect to achieving iron adequacy in toddlers [14].

This approach can also be applied to individual foods/food groups. Quann et al. determined that US children and adults were not achieving the recommended 2.5–3 servings of dairy products per day [12]. Therefore, a theoretical modelling approach was applied to determine the impact on nutrient adequacy if this population were to meet their dairy recommendations. Indeed, the data suggest this would result in a significant reduction in the prevalence of inadequacy of calcium, magnesium and vitamin A [12]. In countries where fortification of milk is not mandatory, an alternate way to achieve nutrient adequacy is through consumption of fortified milk consumed by the family if available in the market. These types of products typically contain additional micronutrients that are often low in the population. A number of recent modelling studies have demonstrated an improvement of micronutrient adequacy following the addition of specially formulated young children- or pre-school children-milks to the diets of these children [9, 11]. Mak et al. found that if Filipino children were to meet the dairy recommendation through adding either a serving of milk or fortified young children milk to their diets, children 1 to 5 years would improve calcium intakes by 30%. Also, the addition of young children milk to the diet reduced the inadequacy of key micronutrients such as iron by 20% and vitamin C by 70% [9]. A similar observation was reported in two studies in young Chinese children, with a reduction of micronutrient inadequacy prevalence after they achieved their dairy recommendation with either milk or a fortified young child milk [15].

The aim of the present study was to determine the role of dairy products and their nutritional impact on Brazilian pre-school children, which has not been thoroughly investigated previously. Key nutrient inadequacies according to dairy product consumption level in Brazilian 4 to 5 years old children were investigated. Furthermore, the study examined the theoretical nutritional impact of increasing consumption of two commonly consumed dairy product in Brazil: cow’s milk and pre-school children milk (PCM), an alternative to cow’s milk that has been demonstrated to improve nutrient intakes and reduce prevalence of nutrient inadequacies in other populations [9, 16, 17].

## Methods

### Study population

The study population included 4 and 5 years old children (*n* = 228) from the Brazilian Kids Nutrition and Health Survey (KNHS) 2019 [7]. KNHS was a cross-sectional survey of Brazilian children 4 to 13 years (*n* = 983) that examined dietary intakes, anthropometric measurements and lifestyle behaviours in three of the most populated regions (Northeast, Southeast, and South), which represent 83.3% of the Brazillian population [7]. A multistage, probability design was applied to ensure representative sampling from the three regions. Parents or caregivers answered age-specific dietary questions, a 24-hour recall, and a general questionnaire on potential influences on the child’s diet including socioeconomics, physical activity, screen time and sleep behavior. Anthropometrics including child height, weight, and waist and hip circumferences were measured at each visit. Data were collected by trained field workers in homes from October 2019 to February 2020. Details on study design, sampling strategy and study approval were published elsewhere [7]. The study was conducted in accordance with the Declaration of Helsinki. Study protocol, procedures, and all materials were reviewed and approved by Institutional Review Boards at RTI International and University of São Paulo. In addition, study approval from CONEP (Comissão Nacional de Ética em Pesquisa) – Brazil’s National Council for Ethics in Research and the advisory body for the Brazilian Ministry of Health [7]. Written consent was obtained from the caregiver prior to data collection.

### Data collection and processing

A 24-hour recall was administered to record all foods and beverages that were consumed by the child on the previous day. Information on the quantity of food consumed, frequencies and location of consumption, types of foods and preparation methods was recorded. A random subset of 25% of the cohort were selected for a second 24-hour recall. Only the first day of recall was used for the current study on dietary modelling. Anthropometric measurements and demographic information were also collected. The energy and nutrient contributions of these foods and beverages consumed were determined using the Nutrition Data System for Research (NDSR) Food and Nutrition Database, adapted for the local region. The total daily intake of 30 nutrients was calculated using the first 24-hour recall. Energy intakes were assessed and participants with energy intakes above or below 3SD of the mean were considered implausible [9, 18]. All recorded foods and beverages were classified into 12 major food groups and appropriate subgroups. The ‘dairy’ food group was further segregated into “milk”, which included fortified and unfortified whole milk (powdered/liquid), “cheese” and “yogurt”. The number of servings of dairy products consumed by each child was calculated to determine adherence to the Brazilian Government recommendation of 2 to 3 servings of dairy products per day [3]. One serving of milk and yogurt is defined as 200 ml and one serving of cheese is defined as 50 g. A dilution factor of 6.7 was applied, based on an average of local powdered milks, to reconstitute powdered milk to liquid milk. Children who consumed no dairy products, or less than two servings per day, were considered as not meeting their dairy recommendations.

### Diet modelling scenarios

Two diet modelling scenarios, encompassing addition and substitution analysis, were applied in the current study. Firstly, the total dairy product consumption of each individual child was calculated. In the first scenario, we added one (200 ml) and two (400 ml) servings of dairy products to each child who was consuming less than one serving of dairy products per day (*n* = 129). Two types of dairy products were tested, 1) unmodified cow’s milk, commonly consumed by children in Brazil, and 2) a widely available pre-school children milk (PCM) for children age 3 years and over, also known as “Dairy Compound” in Brazil. This alternative to cow’s milk has a modified fatty acid profiles and additional micronutrients, typically vitamin D, iron, calcium and zinc, with the aim to supplement micronutrient intakes that are often lacking in the overall diet. Previous studies have demonstrated consumption of fortified young children milk or pre-school children milk can improve nutrient intakes and reduce prevalence of nutrient inadequacies [9, 16, 17]. In scenario two, among children who consumed milk of any kind (fortified and unfortified) (“milk consumers” *n* = 167), we substituted the nutritional composition of their habitual milk with the composition of PCM, volume to volume. The composition of the PCM used in the modelling scenarios as well as unmodified cow’s milk for comparison are reported in Additional Table 1. For both scenarios, total daily nutrient intakes and percentage of nutrient inadequacy (defined using EAR cut-off method, see section Data analysis) before and after applying the scenarios were calculated.

### Data analysis

Data were analyzed using IBM SPSS Statistics for Windows (Version 21), R version × 64.3.6.1 and R-Studio version 1.3.1056. Descriptive statistics of the sample (gender, wealth status and BMI z-score), types of milk and milk products consumed (percentage consumption and amount consumed per day) and whether the children met or did not meet the 2 servings of dairy products per day recommendation. Wealth status was divided into three categories ‘high’ socio economic status (SES), combining groups A, B1, and B2, ‘middle’ SES, grouping children in C1 and C2 groups, and ‘low’ SES combining children from groups D and E [7]. Chi-squared tests were applied to test for significance between socio-demographic characteristics (gender, age, wealth status and BMI z-scores), and whether or not children met dairy recommendations. Chi-squared test was also used to assess significant relationship between the types of dairy products consumed and status of meeting dairy recommendations. Mean nutrient intakes were calculated before and after applying each individual diet modelling scenario. The US Dietary Reference Intakes [19] was used to determine the reference values for nutrient intakes. The acceptable macronutrient distribution ranges (AMDR) were used to evaluate carbohydrates, protein and total fat, as a percentage of total energy (%TE). The estimated average requirements (EAR) cut-off method was applied, where available, to establish adherence to micronutrient recommendations. In the absence of an EAR for a nutrient, the adequate intake (AI) was used as a reference. The proportion of children adhering to the nutrient recommendations was calculated before and after each of the modelling scenarios.

## Results

### Sample characteristics

Of the 228 children included in the study, almost equal split of ages 4-year vs 5-year was observed (Table 1). Fifty-one percent of children were male. Over half of children came from middle SES and 30% from low SES. A quarter of children were classified as obese, 1 in 5 were overweight and almost half were normal weight. No significant difference in age, gender, BMI or wealth status was observed between children who met their dairy recommendations and those who did not.

**Table 1:** Population characteristics (presented as percentage of population)

	Total Population	Not meeting dairy recommendations (< 2 servings/d)	Meeting dairy recommendations (≥2 servings/d)	*P*-value*
	*n* = 228	*n* = 174	*n* = 54	
Gender (%)				
Male	50.9	50.0	53.7	0.634
Female	49.1	50.0	46.3	
Age (%)				
4 years	50.9	54.0	40.7	0.088
5 years	49.1	46.0	59.3	
Wealth Status (%)				
High SES	18.4	17.8	20.4	0.900
Moderate SES	52.6	54.0	48.1	
Low SES	28.9	28.2	31.5	
BMI (z scores)				
Severe Thinness (BMI z-score < −3)	3.1	2.9	3.7	0.923
Thinness (−3 < BMI z-score < −2)	4.4	4.0	5.6	
Normal Weight (−2 < BMI z-score < 1)	47.4	47.1	48.1	
Overweight (1 < BMI z-score < 2)	20.6	24.1	25.9	
Obese (2 < BMI z-score)	24.6	21.8	16.7	

*Chi-square test between meeting dairy recommendations and not meeting dairy recommendations

Categorical variables were presented as % of sample population

Three in four children (*n* = 174) did not meet the Brazilian dairy recommendation of 2 or more servings of dairy products per day (Table 2). Twenty percent of children consumed between 1 and 2 servings per day and 56% consumed less than one serving per day.

**Table 2:** Percentage of dairy consumers and amount consumed per day

		Total Population (*n* = 228)		Not meeting dairy recommendations (< 2 servings/d) (*n* = 174)		Meeting dairy recommendations (≥2 servings/d) (*n* = 54)		*P*-value*
		Consumers (%)	Average amount consumed (g)	Consumers (%)	Average amount consumed (g)	Consumers (%)	Average amount consumed (g)	
Dairy consumption	Whole milk (liquid/powdered)	73.2	147	68.9	118	31.1	217	0.11
	- Fortified	66.60	163	66.5	136	33.5	220	
	- Unfortified	8.30	27	94.7	26	5.3	50	
	Yogurt	19.30	114	84.1	106	15.9	159	
	Cheese	13.20	34	76.7	23	23.3	65	
Dairy recommendations	2+ servings per day	23.8						
	1 to less than 2 servings	20.1						
	< 1 serving per day^a^	56.1						

*Chi-square test between types of dairy products consumed and meeting dairy recommendations and not meeting dairy recommendations

^a^One serving of milk and yogurt is defined as 200 ml and one serving of cheese is defined as 50 g. A dilution factor of 6.7 was applied, based on an average of local powdered milks, to reconstitute powdered milk to liquid milk

Table 3 depicts the mean nutrient intakes of all children and the percentages of children who were below and above the dietary reference intakes. Over a-third of children had inadequate intake of carbohydrate, with energy from fat below the Acceptable Macronutrient Distribution Range, and with inadequate intakes of vitamins A and C. Very high prevalence of inadequate intakes of vitamin D (94%), vitamin E (73%) and calcium (65%) was observed in this sample of Brazilian children.

**Table 3:** Mean nutrient intakes and compliance to dietary reference intakes (DRI) at baseline (*n* = 228)

Nutrient	DRI value			Intake		DRI compliance (%)		
	EAR/ AMDR	AI	UL/ AMDR	Mean	SD	< EAR/ <AMDR	> AI	> UL/ >AMDR
**Macronutrients**								
Energy (kcal/d)	–	–	–	1461	529			
Fat (g/d)	–	–	–	46.4	23.7			
Saturated fat (g/d)	–	–	–	16.9	10.0			
MUFA (% kcal)	–	–	–	8.6	2.8			
PUFA (% kcal)	–	–	–	6.7	2.8			
EicosaPentaenoic Acid (EPA) (g)	–	–	–	0.0	0.0			
DocosaHexaenoic Acid (DHA) (g)	–	–	–	0.0	0.1			
Carbohydrate (g/d)	130	–	–	203.8	76.5	36.0		
Protein (g/d)	19	–	–	59.7	29.3	4.0		
Dietary fiber (g/d)	–	25	–	13.7	6.9		15.0	
Fat (% kcal)	25	–	35	28.1	7.7	35.1		14.9
Saturated fat (% kcal)	–	–	10	10.3	4.1			49.1
Carbohydrate (% kcal)	45	–	65	52.8	8.7	18.0		9.2
Protein (% kcal)	10	–	30	16.3	4.6	4.4		1.3
**Micronutrients**								
Vitamin A (μg retinol activity equivalent/d)	275	–	900	626.4	1293.3	34.6		13.2
Thiamin (mg/d)	0.5	–	–	1.0	0.5	7.9		
Riboflavin (mg/d)	0.5	–	–	1.3	0.7	9.6		
Niacin (mg/d)	6	–	15.0	12.8	7.4	16.7		28.9
Vitamin B-6 (mg/d)	0.5	–	40	1.3	0.7	5.7		0.0
Folate (μg dietary folate equivalents/d)	160	–	400	292.3	139.5	15.8		19.7
Vitamin B-12 (μg/d)	1.0	–	–	4.2	9.4	10.1		
Vitamin C (mg/d)	22	–	650	125.7	519.6	36.0		2.2
Vitamin D (μg/d)	10	–	75	2.8	3.8	94.3		0.0
Vitamin E (mg/d)	6	–	300	5.1	3.4	72.8		0.0
Vitamin K (μg/d)	–	55	–	44.0	31.7		25.9	
Calcium (mg/d)	800	–	2.5	703.8	474.5	65.4		0.9
Iron (mg/d)	4	–	40	11.5	7.7	8.3		0.4
Magnesium (mg/d)	110	–	110	192.9	74.1	8.3		91.7
Phosphorus (mg/d)	405	–	3	893.4	389.5	5.7		0.0
Potassium (mg/d)	–	2.3	–	1729.5	718.8		18.4	
Sodium (mg/d)	–	1000	1900	2034.1	933.2		88.6	53.5
Zinc (mg/d)	4.0	–	12	8.8	5.0	8.3		17.1

*DRI* Dietary reference intake, *EAR* Estimated Average Requirement, *AMDR* Acceptable Macronutrient Distribution Range, *AI* Adequate Intake, *UL* Upper Limit, from the Institute of Medicine

### Scenario 1: Addition of one or two servings of cow’s milk or PCM to the diet of children (*n* = 129)

Among children consuming less than one serving of dairy products at the day of dietary recall, with the addition of one serving of either cow’s milk or PCM, mean energy intakes theoretically increased by approximately 10% and most micronutrient intakes were brought closer to the respective EAR or AI compared to baseline (Table 4). After adding one serving of milk, the proportion of children with inadequacies in vitamin A, E, and calcium decreased by 15%, while the proportion dropped by two-fold in the PCM scenario. In the PCM scenario, all children met the reference intakes for thiamin, vitamin C, iron, phosphorus and zinc. The same trend was observed to a larger extent when adding two servings of milk or PCM (Additional Table 2).

**Table 4:** Scenario 1: Mean nutrient intakes and inadequacy at baseline and after the addition of one serving (200 ml) of milk or PCM to the diet in children with less than one servings of dairy products a day (*n* = 129)

Nutrient				Baseline					Addition of one serving of milk					Addition of one serving of PCM				
	DRI value			Intake		DRI compliance (%)			Intake		DRI compliance (%)			Intake		DRI compliance (%)		
	EAR/ AMDR	AI	UL/ AMDR	Mean	SD	< EAR/ AMDR	> AI	> UL/ AMDR	Mean	SD	< EAR/ AMDR	> AI	> UL/ AMDR	Mean	SD	< EAR/ AMDR	> AI	> UL/ AMDR
**Macronutrients**																		
Energy (kcal/d)	–	–	–	1330	459				1452	459				1473	459			
Fat (g/d)	–	–	–	41.1	21.4				47.6	21.4				47.5	21.4			
MUFA (% kcal)	–	–	–	8.5	2.9				8.8	2.7				9.1	2.6			
PUFA (% kcal)	–	–	–	7.1	3.2				6.7	3.0				7.3	2.9			
Saturated fat (g/d)	–	–	–	13.9	8.1				17.7	8.1				16.1	8.1			
Carbohydrate (g/d)	130	–	–	190.1	66.2	23.0			199.7	66.2	17.0			207.1	66.2	14.0		
Protein (g/d)	19	–	–	52.8	25.5	4.0			59.1	25.5	1.0			57.2	25.5	2.0		
Dietary fiber (g/d)	–	25	–	13.4	6.0		5.0		13.4	6.0		5.0		14.4	6.0		6.0	
Fat (% kcal)	25	–	35	27.2	8.0	42.6		13.2	29.2	7.2	30.2		17.8	28.7	7.1	32.6		14.0
Saturated fat (% kcal)	–	–	10	9.3	4.1			34.1	11.0	3.7			57.4	9.8	3.6			44.2
Carbohydrate (% kcal)	45	–	65	54.1	8.5	15.5		11.6	51.8	7.6	18.6		3.9	53.0	7.5	16.3		4.7
Protein (% kcal)	10	–	30	16.0	4.7	6.2		1.6	16.4	4.3	3.9		1.6	15.6	4.2	5.4		0.8
**Micronutrients**																		
Vitamin A (μg RAE/d)	275	–	900	644.1	1674.0	48.1		12.4	736.4	1674.0	30.2		14.7	801.5	1674.0	17.1		18.6
Thiamin (mg/d)	0.5	–	–	0.9	0.5	11.6			1.0	0.5	6.2			1.3	0.5	0.0		
Riboflavin (mg/d)	0.5	–	–	1.1	0.8	15.5			1.4	0.8	1.6			1.4	0.8	0.8		
Niacin (mg/d)	6	–	15.0	12.2	6.7	14.7		24.8	12.4	6.7	13.2		25.6	14.8	6.7	3.1		41.9
Vitamin B-6 (mg/d)	0.5	–	40	1.2	0.6	7.0		0.0	1.2	0.6	7.0		0.0	1.4	0.6	0.8		0.0
Folate (μg DFE/d)	160	–	400	284.6	139.9	16.3		17.1	294.6	139.9	15.5		19.4	356.9	139.9	3.1		31.8
Vitamin B-12 (μg/d)	1.0	–	–	4.3	12.4	17.1			4.3	12.4	17.1			4.9	12.4	5.4		
Vitamin C (mg/d)	22	–	650	157.5	641.2	37.2		3.1	157.5	641.2	37.2		3.1	189.0	641.2	0.0		3.1
Vitamin D (μg/d)	10	–	75	2.2	2.8	96.9		0.0	2.3	2.8	96.9		0.0	6.0	2.8	93.0		0.0
Vitamin E (mg/d)	6	–	300	4.7	3.2	80.6		0.0	4.8	3.2	79.1		0.0	7.5	3.2	37.2		0.0
Vitamin K (μg/d)	–	55	–	43.9	33.1		24.8		44.5	33.1		24.8		61.2	33.1		50.4	
Calcium (mg/d)	800	–	2.5	486.1	322.1	86.8		0.0	712.1	322.1	69.8		0.0	895.5	322.1	41.1		0.0
Iron (mg/d)	4	–	40	10.8	6.9	9.3		0.0	10.9	6.9	9.3		0.0	17.1	6.9	0.0		0.8
Magnesium (mg/d)	110	–	110	174.3	63.0	13.2		86.8	194.3	63.0	4.7		95.3	189.4	63.0	7.0		93.0
Phosphorus (mg/d)	405	–	3	745.3	296.6	7.8		0.0	913.3	296.6	1.6		0.0	871.3	296.6	1.6		0.0
Potassium (mg/d)	–	2.3	–	1521.0	627.6		10.1		1703.4	627.6		12.4		1521.0	627.6		10.1	
Sodium (mg/d)	–	1000	1900	1939.3	916.9		86.8	48.8	2025.3	916.9		90.7	52.7	2007.0	916.9		90.7	52.7
Zinc (mg/d)	4.0	–	12	7.8	4.6	10.1		9.3	8.5	4.6	7.0		12.4	9.9	4.6	0.0		17.8

*DRI* Dietary reference intake, *EAR* Estimated Average Requirement, *AMDR* Acceptable Macronutrient Distribution Range, *AI* Adequate Intake, *UL* Upper Limit, from the Institute of Medicine

### Scenario 2: Substitution of habitual milk with PCM among milk consumers (*n* = 167)

Children who were milk consumers had average energy intake higher than those who consumed less than one serving of milk (1513 kcal vs. 1330 kcal) (Table 5). Mean macronutrient intakes expressed as percent of total energy (% kcal) were typically within the AMDR, however 40% of children have fat % kcal below the lower AMDR. Greater than 70% of children presented inadequate vitamin D, vitamin E and calcium intakes.

**Table 5:** Scenario 2: Mean nutrient intakes and inadequacy at baseline and after the substitution of usual milk with PCM in ‘milk consumers’ (*n* = 167)

Nutrient				Baseline					Substitution of milk by PCM				
	DRI value			Intake		DRI compliance (%)			Intake		DRI compliance (%)		
	EAR/ AMDR	AI	UL/ AMDR	Mean	SD	< EAR/ AMDR	> AI	> UL/ AMDR	Mean	SD	< EAR/ AMDR	> AI	> UL/ AMDR
**Macronutrients**													
Energy (kcal/d)	–	–	–	1513	524				1544	529			
Fat (g/d)	–	–	–	48.7	22.9				48.3	22.9			
MUFA (% kcal)	–	–	–	18.3	9.7				15.5	9.3			
PUFA (% kcal)	–	–	–	8.8	2.6				9.2	2.6			
Saturated fat (g/d)	–	–	–	18.3	9.7				15.5	9.3			
Carbohydrate (g/d)	130	–	–	208.5	78.5	25.0			220.5	79.7	18.0		
Protein (g/d)	19	–	–	62.9	28.1	0.0			59.5	27.9	0.0		
Dietary fiber (g/d)	–	25	–	13.9	7.1		12.0		15.5	7.1		15.0	
Fat (% kcal)	25	–	35	28.8	7.3	31.1		15.6	27.9	7.1	35.3		15.0
Saturated fat (% kcal)	–	–	10	10.9	3.6			55.7	8.9	3.1			25.7
Carbohydrate (% kcal)	45	–	65	51.8	8.3	19.2		6.0	53.9	7.9	12.0		7.8
Protein (% kcal)	10	–	30	16.7	4.4	1.8		0.6	15.4	4.3	6.6		0.6
**Micronutrients**													
Vitamin A (μg RAE/d)	275	–	900	658.1	1309.4	24.6		14.4	744.8	1319.2	12.6		19.2
Thiamin (mg/d)	0.5	–	–	1.0	0.4	4.8			1.5	0.6	1.8		
Riboflavin (mg/d)	0.5	–	–	1.4	0.7	1.8			1.4	0.7	1.2		
Niacin (mg/d)	6	–	15.0	13.1	7.7	16.8		31.1	17.0	8.3	3.6		50.3
Vitamin B-6 (mg/d)	0.5	–	40	1.4	0.7	4.2		0.0	1.7	0.8	1.2		0.0
Folate (μg DFE/d)	160	–	400	299.4	142.7	15.6		21.6	402.6	155.9	1.2		42.5
Vitamin B-12 (μg/d)	1.0	–	–	4.2	9.5	4.2			3.7	9.5	6.6		
Vitamin C (mg/d)	22	–	650	89.7	312.5	31.1		1.2	138.3	312.8	3.6		1.8
Vitamin D (μg/d)	10	–	75	3.1	4.3	92.8		0.0	8.8	6.7	69.5		0.0
Vitamin E (mg/d)	6	–	300	5.4	3.7	70.1		0.0	9.1	4.5	30.5		0.0
Vitamin K (μg/d)	–	55	–	43.9	31.5		24.6		71.7	36.4		61.7	
Calcium (mg/d)	800	–	2.5	827.4	468.2	56.3		1.2	1092.2	582.5	36.5		1.8
Iron (mg/d)	4	–	40	12.2	8.1	6.6		0.6	21.4	10.8	0.6		4.2
Magnesium (mg/d)	110	–	110	202.9	74.2	5.4		94.6	193.8	73.5	6.6		93.4
Phosphorus (mg/d)	405	–	3	972.3	380.3	1.2		0.0	891.5	363.3	2.4		0.0
Potassium (mg/d)	–	2.3	–	1874.0	703.0		22.2		1412.8	665.6		12.0	
Sodium (mg/d)	–	1000	1900	2049.8	875.4		91.0	55.1	2015.2	879.8		89.2	53.3
Zinc (mg/d)	4.0	–	12	9.4	4.6	4.8		19.8	11.3	5.2	0.6		35.3

*DRI* Dietary reference intake, *EAR* Estimated Average Requirement, *AMDR* Acceptable Macronutrient Distribution Range, *AI* Adequate Intake, *UL* Upper Limit, from the Institute of Medicine

When the habitual milk consumed was theoretically substituted by PCM, the average energy intake rose negligibly to 1544 kcal/day, with little impact on macronutrient AMDR. However, the % kcal from saturated fat reduced while from monounsaturated fat (MUFA) and polyunsaturated fat (PUFA) increased compared to before substitution. Apart from phosphorus and magnesium, the percentage of milk consumers with inadequate intakes dropped for all micronutrients after substitution. In particular, the percentage of inadequate vitamins A, E, K and zinc intakes decreased by two-fold or more, the percentage of calcium and vitamin D inadequacies reduced by 20% and less than 1% of children were below the EAR for iron in this scenario.

## Discussion

Among Brazilian pre-school aged children there is a double public health burden, with both a high prevalence of micronutrient inadequacy combined with around 45% of children being overweight or obese. Inadequate vitamin D, vitamin E and calcium intakes were observed in most of the children. Furthermore, 76% of our sample were not meeting the recommended 2–3 servings of dairy products per day. Interestingly, we found no significant relationship between wealth status and whether children met their dairy recommendation, suggesting dairy consumption may not be linked to affordability. This current study applied a theoretical dietary modelling approach to evaluate if the addition of either cow’s milk or a PCM to the diets of this population would improve micronutrient adequacy. The addition of 2 servings of cow’s milk improved calcium and vitamin A adequacy by close to 40%. While the addition of 2 servings of the PCM led to an amelioration of a number of micronutrient inadequacies, including calcium and a 24% improvement in vitamin D adequacy. The addition of one serving of both cow’s milk and the PCM also improved nutrient adequacy, albeit to a lesser extent. The latter may, however, be a more realistic scenario among children who were receiving less than one serving of dairy products per day. Furthermore, it must be noted that the addition of dairy products resulted in an increase in estimated energy intakes. The second scenario demonstrated the theoretical impact on nutrient adequacy if the current milk consumers were to switch their habitual milk to a PCM. The overall results suggest that such a switch could result in a drastic improvement of adequacy in all vitamins, calcium, iron and zinc, as well as improvement of fatty acid profiles (increased mono- and polyunsaturated fatty acids and reduced saturated fat intakes) and amount (as % kcal) and fibre. Our findings are comparable to similar modelling studies with PCM that recently demonstrated a reduction in key micronutrient inadequacies, including calcium, iron and vitamin D, in UK, Chinese and Filipino children who were not meeting local dairy recommendations [9, 15, 20].

### Impact of increasing dairy consumption on micronutrient adequacy

Low dairy product intakes have been associated with nutrient inadequacies including calcium, magnesium and potassium [21–23]. The Brazilian government recommends that children should have 2–3 serving of dairy products per day [3]. Three out of four children in the current study did not meet this recommendation. Dairy products are an important part of a healthy, balanced diet and provides many key micronutrients, including calcium, which are vital for growth and development [21]. Among children that were consuming dairy products they were typically consuming less than one serving per day, in agreement with low milk consumption in Brazilian adolescents and adults [24]. This study demonstrates the importance of dairy products, including milk and PCM, in supporting children’s improvement of micronutrient adequacy. Previous study has shown that daily consumption of a micronutrient-fortified milk can improve weight-for-eight z-score of toddlers without increasing obesity prevalence, and, unlike unfortified milk, contributed to the reduction of anemia [25]. One exception, although resulting in a very small improvement, is vitamin D. In the current study, even the addition of two servings of PCM in the diet of this population would still leave 3 in 4 children with inadequate vitamin D intakes. Therefore, additional strategies are needed to reduce vitamin D inadequacy in this population. While this study demonstrates that one approach is the inclusion of a fortified PCM in the diets of children, other strategies such as fortification of commonly consumed foods and food groups or adjustment of the national supplementation programme ought to be considered. The Brazilian Society of Paediatricians recommends supplementation for all children under 2 years of age and of any age when belonging to risk groups [26], however this recommendation is not widely followed. Kehoe et al. used dietary modelling to determine the theoretical impact of these strategies: consuming a PCM, consuming cow’s milk fortified with vitamin D or consuming a 5 μg supplement on vitamin D adequacy in Irish pre-school children. All three strategies improved vitamin D adequacy, while a combination approach e.g. fortified milk plus supplement has the greatest impact, reducing inadequacy from 97% to 12–36%, depending on the milk composition [17]. However, the level of vitamin D fortification is important, as countries with mandatory fortification e.g. Canada and the US, still report high levels of vitamin D inadequacy [27–29].

Iron and zinc are key nutrients for growth and development at this stage. Although iron inadequacy is relatively low in this population, a recent publication reports that 1 in 3 Brazilian children are anaemic [28]. This may be due to the high consumption of rice and beans in the Brazilian diet, as these plant-based sources of iron and zinc are not as bioavailable compared to animal food sources [30]. Therefore, the consumption of food and products with a higher iron and zinc bioavailability should be encouraged.

While the addition of dairy products to the diet has a notable impact on micronutrient adequacy, it is important to consider the impact on macronutrient adequacy and energy intakes in light of the high prevalence of overweight and obesity in Brazilian children. Approximately 1 in 5 children who consumed less than one serving of dairy products per day had high energy intakes, and as expected, the theoretical addition of one or two servings of dairy products further increased energy intakes in this population. On the other hand, the substitution of habitual milk with a PCM had little impact on overall energy intake. It is important to consider the overall diets of Brazilian children, where a recent review of the literature suggests that Brazilian children have a high intake of foods that are rich in energy, sugar and fat [31]. This is supported by Horta et al. who reported Brazilian school children have a poor diet quality, as assessed using the Healthy Eating Index-2010, with a high intake of empty calories and inadequate fruit, dairy, wholegrain and vegetables [32]. In the current study there was a 5% higher incidence of obesity in children not meeting dairy recommendations, which may be a result of the displacement of milk with unhealthier foods/beverages. Orden et al. showed, in an Argentine study published in 2019, that low milk consumption was associated with a higher risk of obesity [33]. It is evident that diet quality needs to be improved in this young population to help decrease the prevalence of obesity and micronutrient inadequacies. Dairy products are an important source of many key nutrients that are required for growth and development, therefore children should be encouraged to consume the recommended intake of at least two servings of dairy products per day, as part of a healthy diet, in line with the Brazilian dietary guidelines [3]. Public health strategies are needed to promote a healthy diet in this young population, which could include healthy eating education programmes for parents and children, and the reformulation/fortification of commonly consumed foods or supplementation strategy.

### Strengths and limitations

To our knowledge, it is the first dietary modelling study on Brazilian pre-school children to determine the theoretical impact of milk or PCM consumption on prevalence of micronutrient inadequacy. Due to the high prevalence (45%) of overweight and obesity in this population, the impact on energy intakes and macronutrient adequacy was also described. Dietary modelling has been applied in recent years to determine the impact of making theoretical changes to food or beverages, within the overall diet, on nutrient intakes. While, it is a very useful and scientifically recognised tool, the results must be interpreted with caution as the scenarios were hypothetical and did not take into account the behavioural change that is required to increase dairy products consumption. A limitation is that the focus of the current study is on the dairy food group and its impact on nutrient intakes. An opportunity for further research would be to examine other food groups and whether specific foods and beverages are contributing to high energy and macronutrient intakes, and in turn displacing more healthful food groups. Furthermore, factors related to dietary habits were not collected nor examined in the current study, such as food availability and accessibility, food environment, food cost, culture, and health concerns. The effect of socio-economic status or food affordability was not measured or tested and therefore the study results remain to be theoretical. Further research should collect data on cost of food and better understanding of household food budget, in order to understand how families make decisions around food purchases, and to develop more affordable and nutrient-dense foods for children from lower socio-economic background. It is important to note the small sample size of the current study, and the fact that these children were sampled from three regions of Brazil meant that the results did not represent all pre-school Brazilian population. The dietary data used in the current study came from the first day of the 24-hour recall, which only provides an estimate of the between-person variance and may underestimate consumption of certain nutrients [34]. Hence, the study did not reflect the usual intakes of the children but rather a snapshot of children’s diet for 1 day. Lastly, supplement use was not captured in the current study, therefore the total micronutrient intakes could be underestimated.

In conclusion, using a dietary modelling approach may demonstrate the theoretical benefit of meeting dairy recommendations on micronutrient inadequacy. Calcium, vitamin D and vitamin E are key nutrients of concern in this young Brazilian population. Meeting dairy recommendations with either cow’s milk or a PCM would help to reduce calcium inadequacy, whereas the consumption of a PCM would help reduce inadequacies further for micronutrients such as vitamin D and E. Children should be encouraged to consume two servings of dairy products per day, as part of a healthy balanced diet, and the consumption of fortified foods and beverages or supplements should be encouraged to reduce micronutrient inadequacy in this young population.

## Supplementary Information

**Additional file 1: Additional Table 1.** Composition of one serving (200 ml) of the milk beverages used in the diet modelling scenarios.

**Additional file 2: Additional Table 2** Scenario 1: Mean nutrient intakes and inadequacy at baseline and after the addition of two servings of PCM to the diet in children with less than one serving of dairy product (*n* = 129).

## Data Availability

The datasets generated and/or analysed during the current study are proprietary (e.g., no public funding was used for data collection or analysis), but are available from the corresponding author upon reasonable request.

## Abbreviations

KNHS: Brazil Kids Nutrition and Health Study
PCM: Pre-school Children Milk
CONEP: Comissão Nacional de Ética em Pesquisa
NDSR: Nutrition Data System for Research
SES: Socio Economic Status
AMDR: Acceptable Macronutrient Distribution Range
EAR: Estimated Average Requirement
AI: Adequate Intake
DRI: Dietary reference intake
UL: Upper Limit
MUFA: Monounsaturated Fat
PUFA: Polyunsaturated Fat
