# Supplementary material for: Milk beverages can reduce nutrient inadequacy among Brazilian pre-school children: a dietary modelling study
Source: BMC Nutr. 2022 Nov 1;8:121. doi: 10.1186/s40795-022-00620-w (PMC9623914; doi:10.1186/s40795-022-00620-w)
Supplement: Supplementary file 1 — Additional file 1: Additional Table 1. Composition of one serving (200 ml) of the milk beverages used in the diet modelling scenarios. [file 40795_2022_620_MOESM1_ESM.docx]

**Additional Files**

**Additional Table 1.** Composition of one serving (200ml) of the milk beverages used in the diet modelling scenarios

|  | **Whole milk, not fortified (200ml)** | **Pre-school children milk (PCM) (200ml)** |
| --- | --- | --- |
| **Macronutrients** |  |  |
| Energy (kcal/d) | 128.0 | 143.4 |
| Fat (g/d) | 6.5 | 6.4 |
| Saturated fat (g/d) | 3.7 | 2.2 |
| MUFA (g) | 1.6 | 2.3 |
| PUFA (g) | 0.4 | 1.4 |
| Carbohydrate (g/d) | 9.6 | 17.0 |
| Protein (g/d) | 6.3 | 4.4 |
| Dietary fiber (g/d) | 0.0 | 1.0 |
| **Micronutrients** |  |  |
| Vitamin A (μg retinol activity equivalent/d) | 92.3 | 157.5 |
| Thiamin (mg/d) | 0.1 | 0.3 |
| Riboflavin (mg/d) | 0.3 | 0.4 |
| Niacin (mg/d) | 0.2 | 2.6 |
| Vitamin B-6 (mg/d) | 0.0 | 0.3 |
| Folate (μg dietary folate equivalents/d) | 10.0 | 72.3 |
| Vitamin B-12 (μg/d) | 0.0 | 0.6 |
| Vitamin C (mg/d) | 0.0 | 31.5 |
| Vitamin D (μg/d) | 0.1 | 3.8 |
| Vitamin E (mg/d) | 0.1 | 2.8 |
| Vitamin K (μg/d) | 0.6 | 17.3 |
| Calcium (mg/d) | 226.0 | 409.4 |
| Iron (mg/d) | 0.1 | 6.30 |
| Magnesium (mg/d) | 20.0 | 15.1 |
| Phosphorus (mg/d) | 164.0 | 126.0 |
| Potassium (mg/d) | 182.4 | 0.0 |
| Sodium (mg/d) | 86.0 | 67.7 |
| Zinc (mg/d) | 0.7 | 2.1 |
