# Supplementary material for: Milk beverages can reduce nutrient inadequacy among Brazilian pre-school children: a dietary modelling study
Source: BMC Nutr. 2022 Nov 1;8:121. doi: 10.1186/s40795-022-00620-w (PMC9623914; doi:10.1186/s40795-022-00620-w)
Supplement: Supplementary file 2 — Additional file 2: Additional Table 2 Scenario 1: Mean nutrient intakes and inadequacy at baseline and after the addition of two servings of PCM to the diet in children with less than one serving of dairy product (n = 129). [file 40795_2022_620_MOESM2_ESM.docx]

**Additional Table 2** Scenario 1: Mean nutrient intakes and inadequacy at baseline and after the addition of two servings of PCM to the diet in children with less than one serving of dairy (n=129)

|  |  |  |  | **Baseline** | | | | | **Addition of two servings of milk** | | | | | | **Addition of two servings of PCM** | | | | | | |
| --- | --- | --- | --- | --- | --- | --- | --- | --- | --- | --- | --- | --- | --- | --- | --- | --- | --- | --- | --- | --- | --- |
|  | **DRI value** | | | **Intake** | | **DRI compliance (%)** | | | **Intake** | | **DRI compliance (%)** | | | | **Intake** | | | **DRI compliance (%)** | | | |
| **Nutrient** | **EAR/ AMDR** | **AI** | **UL/**  **AMDR** | **Mean** | **SD** | **< EAR/ AMDR** | **> AI** | **> UL/ AMDR** | **Mean** | **SD** | **< EAR/ AMDR** | **> AI** | **> UL/ AMDR** | **Mean** | | **SD** | **< EAR/ AMDR** | | **> AI** | **> UL/ AMDR** |  |
| **Macronutrients** |  |  |  |  |  |  |  |  |  |  |  |  |  |  | |  |  | |  |  |  |
| Energy (kcal/d) | – | – | – | 1330 | 459 |  |  |  | 1574 | 459 |  |  |  | 1617 | | 459 |  | |  |  |  |
| Fat (g/d) | – | – | – | 41.1 | 21.4 |  |  |  | 54.1 | 21.4 |  |  |  | 53.9 | | 21.4 |  | |  |  |  |
| MUFA (%kcal) | – | – | – | 13.9 | 8.1 |  |  |  | 21.4 | 8.1 |  |  |  | 18.3 | | 8.1 |  | |  |  |  |
| PUFA (%kcal) | – | – | – | 8.5 | 2.9 |  |  |  | 9.1 | 2.4 |  |  |  | 9.6 | | 2.3 |  | |  |  |  |
| Saturated fat (g/d) | – | – | – | 7.1 | 3.2 |  |  |  | 6.4 | 2.7 |  |  |  | 7.5 | | 2.6 |  | |  |  |  |
| Carbohydrate (g/d) | 130 | – | – | 190.1 | 66.2 | 23.0 |  |  | 209.3 | 66.2 | 13.0 |  |  | 224.1 | | 66.2 | 4.0 | |  |  |  |
| Protein (g/d) | 19 | – | – | 52.8 | 25.5 | 4.0 |  |  | 65.4 | 25.5 | 0.0 |  |  | 61.6 | | 25.5 | 0.0 | |  |  |  |
| Dietary fiber (g/d) | – | 25 | – | 13.4 | 6.0 |  | 5.0 |  | 13.4 | 6.0 |  | 5.0 |  | 15.3 | | 6.0 |  | | 7.0 |  |  |
| Fat (% kcal) | 25 | – | 35 | 27.2 | 8.0 | 42.6 |  | 13.2 | 30.8 | 6.6 | 18.6 |  | 23.3 | 29.8 | | 6.4 | 23.3 | |  | 17.1 |  |
| Saturated fat (% kcal) | – | – | 10 | 9.3 | 4.1 |  |  | 34.1 | 12.4 | 3.4 |  |  | 77.5 | 10.2 | | 3.3 |  | |  | 46.5 |  |
| Carbohydrate (% kcal) | 45 | – | 65 | 54.1 | 8.5 | 15.5 |  | 11.6 | 49.9 | 7.0 | 26.4 |  | 1.6 | 52.2 | | 6.8 | 16.3 | |  | 2.3 |  |
| Protein (% kcal) | 10 | – | 30 | 16.0 | 4.7 | 6.2 |  | 1.6 | 16.7 | 3.9 | 1.6 |  | 0.8 | 15.2 | | 3.8 | 4.7 | |  | 0.8 |  |
| **Micronutrients** |  |  |  |  |  |  |  |  |  |  |  |  |  |  | |  |  | |  |  |  |
| Vitamin A (μg RAE/d) | 275 | – | 900 | 644.1 | 1674.0 | 48.1 |  | 12.4 | 828.7 | 1674.0 | 10.9 |  | 19.4 | 959.0 | | 1674.0 | 0.0 | |  | 20.9 |  |
| Thiamin (mg/d) | 0.5 | – | – | 0.9 | 0.5 | 11.6 |  |  | 1.1 | 0.5 | 3.1 |  |  | 1.6 | | 0.5 | 0.0 | |  |  |  |
| Riboflavin (mg/d) | 0.5 | – | – | 1.1 | 0.8 | 15.5 |  |  | 1.7 | 0.8 | 0.0 |  |  | 1.8 | | 0.8 | 0.0 | |  |  |  |
| Niacin (mg/d) | 6 | – | 15.0 | 12.2 | 6.7 | 14.7 |  | 24.8 | 12.6 | 6.7 | 12.4 |  | 26.4 | 17.3 | | 6.7 | 0.0 | |  | 55.8 |  |
| Vitamin B-6 (mg/d) | 0.5 | – | 40 | 1.2 | 0.6 | 7.0 |  | 0.0 | 1.2 | 0.6 | 5.4 |  | 0.0 | 1.7 | | 0.6 | 0.0 | |  | 0.0 |  |
| Folate (μg DFE/d) | 160 | – | 400 | 284.6 | 139.9 | 16.3 |  | 17.1 | 304.6 | 139.9 | 13.2 |  | 19.4 | 429.1 | | 139.9 | 0.0 | |  | 53.5 |  |
| Vitamin B-12 (μg/d) | 1.0 | – | – | 4.3 | 12.4 | 17.1 |  |  | 4.3 | 12.4 | 17.1 |  |  | 5.5 | | 12.4 | 0.0 | |  |  |  |
| Vitamin C (mg/d) | 22 | – | 650 | 157.5 | 641.2 | 37.2 |  | 3.1 | 157.5 | 641.2 | 37.2 |  | 3.1 | 220.5 | | 641.2 | 0.0 | |  | 3.1 |  |
| Vitamin D (μg/d) | 10 | – | 75 | 2.2 | 2.8 | 96.9 |  | 0.0 | 2.4 | 2.8 | 96.9 |  | 0.0 | 9.8 | | 2.8 | 72.9 | |  | 0.0 |  |
| Vitamin E (mg/d) | 6 | – | 300 | 4.7 | 3.2 | 80.6 |  | 0.0 | 4.9 | 3.2 | 78.3 |  | 0.0 | 10.3 | | 3.2 | 0.0 | |  | 0.0 |  |
| Vitamin K (μg/d) | – | 55 | – | 43.9 | 33.1 |  | 24.8 |  | 45.1 | 33.1 |  | 27.1 |  | 78.5 | | 33.1 |  | | 75.2 |  |  |
| Calcium (mg/d) | 800 | – | 2.5 | 486.1 | 322.1 | 86.8 |  | 0.0 | 938.1 | 322.1 | 37.2 |  | 0.0 | 1304.9 | | 322.1 | 0.0 | |  | 0.8 |  |
| Iron (mg/d) | 4 | – | 40 | 10.8 | 6.9 | 9.3 |  | 0.0 | 11.0 | 6.9 | 8.5 |  | 0.0 | 23.4 | | 6.9 | 0.0 | |  | 2.3 |  |
| Magnesium (mg/d) | 110 | – | 110 | 174.3 | 63.0 | 13.2 |  | 86.8 | 214.3 | 63.0 | 1.6 |  | 98.4 | 204.5 | | 63.0 | 2.3 | |  | 97.7 |  |
| Phosphorus (mg/d) | 405 | – | 3 | 745.3 | 296.6 | 7.8 |  | 0.0 | 1081.3 | 296.6 | 0.0 |  | 0.0 | 997.2 | | 296.6 | 0.8 | |  | 0.0 |  |
| Potassium (mg/d) | – | 2.3 | – | 1521.0 | 627.6 |  | 10.1 |  | 1885.8 | 627.6 |  | 24.0 |  | 1521.0 | | 627.6 |  | | 10.1 |  |  |
| Sodium (mg/d) | – | 1 | 1.9 | 1939.3 | 916.9 |  | 86.8 | 48.8 | 2111.3 | 916.9 |  | 91.5 | 55.0 | 2074.7 | | 916.9 |  | | 91.5 | 53.5 |  |
| Zinc (mg/d) | 4.0 | – | 12 | 7.8 | 4.6 | 10.1 |  | 9.3 | 9.3 | 4.6 | 3.9 |  | 14.7 | 11.9 | | 4.6 | 0.0 | |  | 41.1 |  |

Dietary reference intake (DRI), Estimated Average Requirement (EAR)**,** Acceptable Macronutrient Distribution Range (AMDR), Adequate Intake (AI), Upper Limit (UL) from the Institute of Medicine.
